# (5-Bromo-2-methyl­phen­yl)(4-eth­oxy­phen­yl)methanone

**DOI:** 10.1107/S1600536810027327

**Published:** 2010-07-17

**Authors:** Yong-Heng Shi, Gui-Long Zhao, Hua Shao, Wei Liu, Wei-Ren Xu

**Affiliations:** a Tianjin Key Laboratory of Molecular Design and Drug Discovery, Tianjin Institute of Pharmaceutical Research, Tianjin 300193, People’s Republic of China; b School of Pharmacy, Tianjin Medical University, Tianjin 300070, People’s Republic of China

## Abstract

In the title compound, C_16_H_15_BrO_2_, the dihedral angle between the benzene rings is 68.5 (2)°. In the crystal structure, mol­ecules are linked by weak C—H⋯O hydrogen bonds into chains parallel to the *b* axis.

## Related literature

For details of the biological activity of SGLT2 inhibitors, see: Meng *et al.* (2008 ▶). For bond-length data, see: Allen *et al.* (1987 ▶).
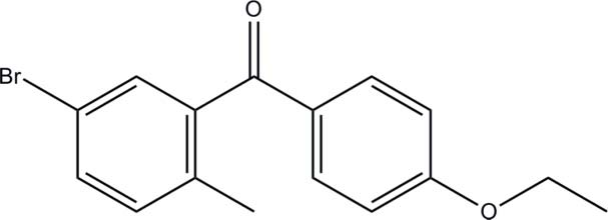


## Experimental

### 

#### Crystal data

C_16_H_15_BrO_2_
*M* *_r_* = 319.19
Orthorhombic, 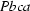

*a* = 9.5730 (19) Å
*b* = 13.188 (3) Å
*c* = 22.205 (4) Å
*V* = 2803.4 (10) Å^3^
*Z* = 8
Mo *K*α radiation
μ = 2.93 mm^−1^
*T* = 113 K
0.30 × 0.20 × 0.16 mm

#### Data collection

Rigaku Saturn CCD area-detector diffractometer
Absorption correction: multi-scan (*CrystalClear*; Rigaku, 2005 ▶) *T*_min_ = 0.474, *T*_max_ = 0.652
17506 measured reflections
2479 independent reflections
2133 reflections with *I* > 2σ(*I*)
*R*_int_ = 0.044

#### Refinement

*R*[*F* ^2^ > 2σ(*F* ^2^)] = 0.041
*wR*(*F* ^2^) = 0.107
*S* = 1.10
2479 reflections
175 parameters
H-atom parameters constrained
Δρ_max_ = 0.84 e Å^−3^
Δρ_min_ = −0.57 e Å^−3^

### 

Data collection: *CrystalClear* (Rigaku, 2005 ▶); cell refinement: *CrystalClear*; data reduction: *CrystalClear*; program(s) used to solve structure: *SHELXTL* (Sheldrick, 2008 ▶); program(s) used to refine structure: *SHELXTL*; molecular graphics: *SHELXTL*; software used to prepare material for publication: *SHELXTL*.

## Supplementary Material

Crystal structure: contains datablocks I, global. DOI: 10.1107/S1600536810027327/rz2475sup1.cif

Structure factors: contains datablocks I. DOI: 10.1107/S1600536810027327/rz2475Isup2.hkl

Additional supplementary materials:  crystallographic information; 3D view; checkCIF report

## Figures and Tables

**Table 1:** Hydrogen-bond geometry (Å, °)

*D*—H⋯*A*	*D*—H	H⋯*A*	*D*⋯*A*	*D*—H⋯*A*
C11—H11⋯O1^i^	0.95	2.42	3.313 (3)	156

Symmetry code: (i) 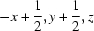.

**Fig. 1.**
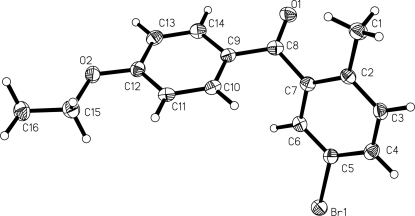
The molecular structure of the title compound, with displacement ellipsoids drawn at the 40% probability level.

## supplementary crystallographic information

### Comment

Dapagliflozin is an anti-diabetic agent through the inhibition of renal SGLT2, which was developed by Bristol-Myers Squibb Company and is now in the phase III clinical trial (Meng *et al.*, 2008). During the discovery of our own SGLT2 inhibitors as anti-diabetic agents, we prepared the derivatives of dapagliflozin (Meng *et al.*, 2008) for biological evaluation, and the title compound, (5-bromo-2-methylphenyl)(4-ethoxyphenyl)methanone, was prepared as an important intermediate.

In title compound, C_16_H_15_BrO_2_, bond lengths are normal (Allen *et al.*, 1987)). The dihedral angle between the benzene rings (C2—C7 and C9—C14) is 68.5 (2)°. In the crystal structure, molecules interact through weak C—H···O hydrogen bonds to form chains parallel to the *b* axis.

### Experimental

A round-bottomed flask was charged with 2.15 g (10 mmol) of 5-bromo-2-methylbenoic acid, 1 drop of DMF, 1.27 g (10 mmol) of oxalyl chloride and 3 ml of dried dichloromethane, and the mixture was stirred at room temperature over night until a clear solution formed. The reaction mixture was evaporated on a rotary evaporator to give crude 5-bromo-2-chlorobenzoyl chloride, which was dissolved in 15 ml of dried dichloromethane. The solution thus obtained was stirred while being cooled with an ice-salt bath, and 1.22 g (10 mmol) of phenetole was added followed by the addition of 1.60 g (12 mmol) of anhydrous aluminium chloride in a portionwise manner. The resulting mixture was stirred at this temperature for 1 h and poured into 150 ml of ice-water. The mixture formed was extracted with three 50 ml portions of dichloromethane, and the combined extracts were washed with saturated brine, dried over sodium sulfate and evaporated on a rotary evaporator to afford the crude title compound. Pure title compound was obtained by column chromatography. Crystals suitable for X-ray diffraction were obtained through slow evaporation of a solution of the pure title compound in ethyl acetate/petroleum ether (1/5 *v*/*v*).

### Refinement

All H atoms were found on difference Fourier maps, and included in the final cycles of refinement using a riding model, with C—H = 0.95–0.99 Å and with *U*_iso_(H) = 1.2*U*_eq_(C) for aryl and methylene H atoms and 1.5*U*_eq_(C) for the methyl H atoms.

### Crystal data

**Table:**

C_16_H_15_BrO_2_	*F*(000) = 1296
*M**_r_* = 319.19	*D*_x_ = 1.513 Mg m^−^^3^
Orthorhombic, *P**b**c**a*	Mo *K*α radiation, λ = 0.71073 Å
Hall symbol: -P 2ac 2ab	Cell parameters from 6268 reflections
*a* = 9.5730 (19) Å	θ = 2.1–27.9°
*b* = 13.188 (3) Å	µ = 2.93 mm^−^^1^
*c* = 22.205 (4) Å	*T* = 113 K
*V* = 2803.4 (10) Å^3^	Block, colorless
*Z* = 8	0.30 × 0.20 × 0.16 mm

### Data collection

**Table:**

Rigaku Saturn CCD area-detector diffractometer	2479 independent reflections
Radiation source: rotating anode	2133 reflections with *I* > 2σ(*I*)
confocal	*R*_int_ = 0.044
Detector resolution: 7.31 pixels mm^-1^	θ_max_ = 25.0°, θ_min_ = 1.8°
ω and φ scans	*h* = −11→10
Absorption correction: multi-scan (*CrystalClear*; Rigaku, 2005)	*k* = −15→15
*T*_min_ = 0.474, *T*_max_ = 0.652	*l* = −26→19
17506 measured reflections	

### Refinement

**Table:**

Refinement on *F*^2^	Secondary atom site location: difference Fourier map
Least-squares matrix: full	Hydrogen site location: inferred from neighbouring sites
*R*[*F*^2^ > 2σ(*F*^2^)] = 0.041	H-atom parameters constrained
*wR*(*F*^2^) = 0.107	*w* = 1/[σ^2^(*F*_o_^2^) + (0.0598*P*)^2^ + 1.3319*P*] where *P* = (*F*_o_^2^ + 2*F*_c_^2^)/3
*S* = 1.10	(Δ/σ)_max_ = 0.004
2479 reflections	Δρ_max_ = 0.84 e Å^−^^3^
175 parameters	Δρ_min_ = −0.57 e Å^−^^3^
0 restraints	Extinction correction: *SHELXTL* (Sheldrick, 2008), Fc^*^=kFc[1+0.001xFc^2^λ^3^/sin(2θ)]^-1/4^
Primary atom site location: structure-invariant direct methods	Extinction coefficient: 0.0120 (8)

### Special details

**Table:**

Geometry. All e.s.d.'s (except the e.s.d. in the dihedral angle between two l.s. planes) are estimated using the full covariance matrix. The cell e.s.d.'s are taken into account individually in the estimation of e.s.d.'s in distances, angles and torsion angles; correlations between e.s.d.'s in cell parameters are only used when they are defined by crystal symmetry. An approximate (isotropic) treatment of cell e.s.d.'s is used for estimating e.s.d.'s involving l.s. planes.
Refinement. Refinement of *F*^2^ against ALL reflections. The weighted *R*-factor *wR* and goodness of fit *S* are based on *F*^2^, conventional *R*-factors *R* are based on *F*, with *F* set to zero for negative *F*^2^. The threshold expression of *F*^2^ > σ(*F*^2^) is used only for calculating *R*-factors(gt) *etc*. and is not relevant to the choice of reflections for refinement. *R*-factors based on *F*^2^ are statistically about twice as large as those based on *F*, and *R*- factors based on ALL data will be even larger.

### Fractional atomic coordinates and isotropic or equivalent isotropic displacement parameters (Å^2^)

**Table:**

	*x*	*y*	*z*	*U*_iso_*/*U*_eq_	
Br1	−0.00393 (3)	0.34535 (2)	0.307507 (13)	0.03274 (18)	
O1	0.0807 (2)	0.03567 (15)	0.10091 (9)	0.0353 (5)	
O2	0.1222 (2)	0.43270 (15)	−0.05895 (8)	0.0322 (5)	
C1	0.3438 (3)	0.0117 (2)	0.18087 (13)	0.0354 (7)	
H1A	0.4351	0.0104	0.2009	0.053*	
H1B	0.3562	0.0299	0.1384	0.053*	
H1C	0.3005	−0.0555	0.1836	0.053*	
C2	0.2512 (3)	0.08893 (19)	0.21113 (13)	0.0259 (6)	
C3	0.2670 (3)	0.1078 (2)	0.27228 (13)	0.0286 (6)	
H3	0.3328	0.0691	0.2946	0.034*	
C4	0.1895 (3)	0.1816 (2)	0.30181 (12)	0.0303 (7)	
H4	0.1999	0.1921	0.3439	0.036*	
C5	0.0968 (3)	0.2395 (2)	0.26867 (11)	0.0269 (6)	
C6	0.0771 (3)	0.2229 (2)	0.20780 (12)	0.0264 (6)	
H6	0.0129	0.2633	0.1857	0.032*	
C7	0.1528 (3)	0.1459 (2)	0.17905 (12)	0.0255 (6)	
C8	0.1169 (3)	0.1222 (2)	0.11452 (12)	0.0267 (6)	
C9	0.1212 (3)	0.2048 (2)	0.06972 (11)	0.0246 (6)	
C10	0.2006 (3)	0.2915 (2)	0.07938 (11)	0.0256 (6)	
H10	0.2518	0.2979	0.1158	0.031*	
C11	0.2069 (3)	0.3687 (2)	0.03726 (11)	0.0250 (6)	
H11	0.2634	0.4268	0.0442	0.030*	
C12	0.1289 (3)	0.3597 (2)	−0.01543 (12)	0.0267 (6)	
C13	0.0496 (3)	0.2727 (2)	−0.02606 (12)	0.0308 (7)	
H13	−0.0032	0.2668	−0.0621	0.037*	
C14	0.0480 (3)	0.1956 (2)	0.01559 (12)	0.0294 (6)	
H14	−0.0034	0.1356	0.0075	0.035*	
C15	0.2006 (3)	0.5248 (2)	−0.04965 (13)	0.0334 (7)	
H15A	0.3020	0.5102	−0.0497	0.040*	
H15B	0.1756	0.5557	−0.0105	0.040*	
C16	0.1645 (3)	0.5955 (2)	−0.10032 (13)	0.0364 (7)	
H16A	0.1906	0.5644	−0.1388	0.055*	
H16B	0.2154	0.6594	−0.0952	0.055*	
H16C	0.0638	0.6090	−0.1000	0.055*	

### Atomic displacement parameters (Å^2^)

**Table:**

	*U*^11^	*U*^22^	*U*^33^	*U*^12^	*U*^13^	*U*^23^
Br1	0.0346 (3)	0.0327 (3)	0.0309 (3)	0.00282 (11)	0.01170 (11)	−0.00058 (11)
O1	0.0412 (13)	0.0268 (11)	0.0379 (11)	−0.0026 (9)	−0.0050 (9)	−0.0040 (8)
O2	0.0373 (12)	0.0317 (12)	0.0276 (10)	−0.0050 (9)	−0.0020 (8)	0.0032 (8)
C1	0.0350 (17)	0.0322 (18)	0.0390 (16)	0.0046 (14)	−0.0043 (13)	−0.0038 (13)
C2	0.0237 (15)	0.0226 (15)	0.0314 (15)	−0.0026 (12)	−0.0001 (11)	0.0005 (11)
C3	0.0281 (16)	0.0264 (16)	0.0314 (15)	0.0010 (12)	−0.0050 (11)	0.0036 (11)
C4	0.0317 (16)	0.0343 (16)	0.0248 (14)	−0.0063 (13)	0.0020 (11)	0.0019 (11)
C5	0.0265 (15)	0.0269 (15)	0.0273 (14)	−0.0050 (12)	0.0061 (11)	0.0013 (11)
C6	0.0209 (14)	0.0275 (15)	0.0307 (14)	0.0008 (11)	0.0022 (11)	0.0026 (11)
C7	0.0225 (15)	0.0246 (16)	0.0293 (14)	−0.0032 (11)	−0.0001 (11)	−0.0010 (10)
C8	0.0180 (14)	0.0304 (16)	0.0318 (15)	0.0022 (11)	0.0000 (10)	−0.0044 (13)
C9	0.0207 (14)	0.0278 (15)	0.0254 (14)	0.0031 (11)	−0.0018 (10)	−0.0037 (11)
C10	0.0197 (14)	0.0326 (16)	0.0245 (13)	0.0032 (12)	−0.0018 (10)	−0.0054 (11)
C11	0.0199 (14)	0.0273 (14)	0.0278 (14)	−0.0022 (11)	0.0023 (10)	−0.0060 (11)
C12	0.0245 (15)	0.0302 (16)	0.0254 (14)	0.0011 (12)	0.0029 (11)	0.0004 (11)
C13	0.0298 (16)	0.0361 (18)	0.0266 (15)	−0.0041 (13)	−0.0052 (12)	−0.0055 (12)
C14	0.0253 (15)	0.0309 (16)	0.0321 (16)	−0.0037 (13)	−0.0017 (12)	−0.0033 (12)
C15	0.0311 (16)	0.0324 (17)	0.0367 (16)	−0.0045 (13)	0.0001 (12)	0.0007 (12)
C16	0.0335 (17)	0.0361 (18)	0.0397 (16)	−0.0011 (13)	0.0051 (13)	0.0063 (13)

### Geometric parameters (Å, °)

**Table:**

Br1—C5	1.903 (3)	C8—C9	1.475 (4)
O1—C8	1.230 (3)	C9—C10	1.389 (4)
O2—C12	1.365 (3)	C9—C14	1.397 (4)
O2—C15	1.442 (3)	C10—C11	1.385 (4)
C1—C2	1.509 (4)	C10—H10	0.9500
C1—H1A	0.9800	C11—C12	1.393 (4)
C1—H1B	0.9800	C11—H11	0.9500
C1—H1C	0.9800	C12—C13	1.396 (4)
C2—C3	1.389 (4)	C13—C14	1.374 (4)
C2—C7	1.400 (4)	C13—H13	0.9500
C3—C4	1.388 (4)	C14—H14	0.9500
C3—H3	0.9500	C15—C16	1.502 (4)
C4—C5	1.382 (4)	C15—H15A	0.9900
C4—H4	0.9500	C15—H15B	0.9900
C5—C6	1.382 (4)	C16—H16A	0.9800
C6—C7	1.401 (4)	C16—H16B	0.9800
C6—H6	0.9500	C16—H16C	0.9800
C7—C8	1.506 (4)		
			
C12—O2—C15	117.9 (2)	C10—C9—C8	121.2 (2)
C2—C1—H1A	109.5	C14—C9—C8	120.2 (2)
C2—C1—H1B	109.5	C11—C10—C9	121.7 (2)
H1A—C1—H1B	109.5	C11—C10—H10	119.2
C2—C1—H1C	109.5	C9—C10—H10	119.2
H1A—C1—H1C	109.5	C10—C11—C12	118.8 (3)
H1B—C1—H1C	109.5	C10—C11—H11	120.6
C3—C2—C7	118.3 (2)	C12—C11—H11	120.6
C3—C2—C1	119.5 (2)	O2—C12—C11	124.0 (2)
C7—C2—C1	122.1 (3)	O2—C12—C13	115.7 (2)
C2—C3—C4	122.0 (3)	C11—C12—C13	120.2 (2)
C2—C3—H3	119.0	C14—C13—C12	120.0 (3)
C4—C3—H3	119.0	C14—C13—H13	120.0
C5—C4—C3	118.6 (2)	C12—C13—H13	120.0
C5—C4—H4	120.7	C13—C14—C9	120.6 (3)
C3—C4—H4	120.7	C13—C14—H14	119.7
C6—C5—C4	121.4 (3)	C9—C14—H14	119.7
C6—C5—Br1	119.3 (2)	O2—C15—C16	107.2 (2)
C4—C5—Br1	119.3 (2)	O2—C15—H15A	110.3
C5—C6—C7	119.3 (3)	C16—C15—H15A	110.3
C5—C6—H6	120.4	O2—C15—H15B	110.3
C7—C6—H6	120.4	C16—C15—H15B	110.3
C2—C7—C6	120.4 (3)	H15A—C15—H15B	108.5
C2—C7—C8	121.7 (2)	C15—C16—H16A	109.5
C6—C7—C8	117.8 (2)	C15—C16—H16B	109.5
O1—C8—C9	121.8 (2)	H16A—C16—H16B	109.5
O1—C8—C7	119.4 (2)	C15—C16—H16C	109.5
C9—C8—C7	118.8 (2)	H16A—C16—H16C	109.5
C10—C9—C14	118.6 (3)	H16B—C16—H16C	109.5
			
C7—C2—C3—C4	−0.7 (4)	O1—C8—C9—C10	−159.8 (3)
C1—C2—C3—C4	176.7 (3)	C7—C8—C9—C10	22.6 (4)
C2—C3—C4—C5	−1.8 (4)	O1—C8—C9—C14	18.3 (4)
C3—C4—C5—C6	2.2 (4)	C7—C8—C9—C14	−159.3 (3)
C3—C4—C5—Br1	−177.0 (2)	C14—C9—C10—C11	0.9 (4)
C4—C5—C6—C7	−0.1 (4)	C8—C9—C10—C11	179.0 (2)
Br1—C5—C6—C7	179.1 (2)	C9—C10—C11—C12	1.5 (4)
C3—C2—C7—C6	2.8 (4)	C15—O2—C12—C11	−0.1 (4)
C1—C2—C7—C6	−174.6 (3)	C15—O2—C12—C13	179.1 (2)
C3—C2—C7—C8	−172.7 (2)	C10—C11—C12—O2	177.1 (2)
C1—C2—C7—C8	10.0 (4)	C10—C11—C12—C13	−2.0 (4)
C5—C6—C7—C2	−2.4 (4)	O2—C12—C13—C14	−179.0 (3)
C5—C6—C7—C8	173.2 (2)	C11—C12—C13—C14	0.2 (4)
C2—C7—C8—O1	53.5 (4)	C12—C13—C14—C9	2.2 (4)
C6—C7—C8—O1	−122.1 (3)	C10—C9—C14—C13	−2.8 (4)
C2—C7—C8—C9	−128.8 (3)	C8—C9—C14—C13	179.1 (3)
C6—C7—C8—C9	55.6 (3)	C12—O2—C15—C16	−174.5 (2)

### Hydrogen-bond geometry (Å, °)

**Table:**

*D*—H···*A*	*D*—H	H···*A*	*D*···*A*	*D*—H···*A*
C11—H11···O1^i^	0.95	2.42	3.313 (3)	156

Symmetry codes: (i) −*x*+1/2, *y*+1/2, *z*.
